# Digital image analysis and artificial intelligence in pathology diagnostics—the Swiss view

**DOI:** 10.1007/s00292-023-01262-w

**Published:** 2023-11-21

**Authors:** Sabina Berezowska, Gieri Cathomas, Rainer Grobholz, Maurice Henkel, Wolfram Jochum, Viktor H. Koelzer, Mario Kreutzfeldt, Kirsten D. Mertz, Matthias Rössle, Davide Soldini, Inti Zlobec, Andrew Janowczyk

**Affiliations:** 1 Institut Universitaire de Pathologie, Centre Hospitalier Universitaire Vaudois (CHUV), Rue du Bugnon 25, 1011 Lausanne, Switzerland; 2 Institute of Tissue Medicine and Pathology, University of Bern, Murtenstr. 31, 3008 Bern, Switzerland; 3 Medical Faculty, University of Zurich, Zurich, Switzerland; 4 Institute of Pathology, Cantonal Hospital Aarau, Aarau, Switzerland; 5 Research & Analytic Services, University Hospital Basel, Basel, Switzerland; 6 Institute of Radiology, University Hospital Basel, Basel, Switzerland; 7 University Basel, Basel, Switzerland; 8 Institute of Pathology, Cantonal Hospital St. Gallen, 9007 St. Gallen, Switzerland; 9 Department of Pathology and Molecular Pathology, University and University Hospital of Zurich, Zurich, Switzerland; 10 Department of Pathology and Immunology, University of Geneva, Geneva, Switzerland; 11 Division of Clinical Pathology, Geneva University Hospital, Geneva, Switzerland; 12 Institute of Pathology, Cantonal Hospital Baselland, Liestal, Switzerland; 13 Pathologie, Luzerner Kantonsspital, Spitalstr., 6000 Luzern 16, Switzerland; 14 Pathologie Zentrum Zürich medica, Hottingerstr. 9, 8024 Zürich, Switzerland; 15 Institute of Tissue Medicine and Pathology, University of Bern, Murtenstr. 31, 3008 Bern, Switzerland; 16 Department of Biomedical Engineering, Emory University, Atlanta, USA; 17 Department of Oncology, Division of Precision Oncology, Geneva University Hospitals, Geneva, Switzerland; 18 Department of Diagnostics, Division of Clinical Pathology, Geneva University Hospitals, Geneva, Switzerland

**Keywords:** Pathology, Digitalization, Image analysis, Artificial intelligence, Delphi process, Pathologie, Digititalisierung, Bildanalyse, Künstliche Intelligenz, Delphi Befragung

## Abstract

Digital pathology (DP) is increasingly entering routine clinical pathology diagnostics. As digitization of the routine caseload advances, implementation of digital image analysis algorithms and artificial intelligence tools becomes not only attainable, but also desirable in daily sign out. The Swiss Digital Pathology Consortium (SDiPath) has initiated a Delphi process to generate best-practice recommendations for various phases of the process of digitization in pathology for the local Swiss environment, encompassing the following four topics: i) scanners, quality assurance, and validation of scans; ii) integration of scanners and systems into the pathology laboratory information system; iii) the digital workflow; and iv) digital image analysis (DIA)/artificial intelligence (AI). The current article focuses on the DIA-/AI-related recommendations generated and agreed upon by the working group and further verified by the Delphi process among the members of SDiPath. Importantly, they include the view and the currently perceived needs of practicing pathologists from multiple academic and cantonal hospitals as well as private practices.

## Abstract (de)

Digitale Pathologe (DP) wird zunehmend in der Routinediagnostik der klinischen Pathologie eingesetzt. Mit fortschreitender Digitalisierung der Routinefälle wird die Implementierung digitaler Bildanalysealgorithmen und künstlicher Intelligenz nicht nur machbar, sondern auch für die tägliche Praxis wünschenswert. Das Schweizer Konsortium für Digitale Pathologie (Swiss Digital Pathology Consortium, SDiPath) hat einen Delphi-Prozess initiiert, um Empfehlungen für das beste Vorgehen in Bezug auf verschiedene Phasen des Digitalisierungsprozesses in der Pathologie für die lokalen Verhältnisse in der Schweiz zu erstellen, dazu gehören die folgenden 4 Themen: i) Scanner, Qualitätssicherung und Validierung der eingescannten Schnittpräparate; ii) Integration von Scannern und Systemen in das Laborinformationssystem; iii) der digitale Arbeitsablauf; und iv) digitale Bildanalyse (DIA)/künstliche Intelligenz (KI). Im vorliegenden Beitrag liegt der Schwerpunkt auf den Empfehlungen hinsichtlich DIA und KI, die von einer Arbeitsgruppe erarbeitet und mittels Delphi-Prozess durch die Mitglieder des SDiPath-Konsortiums bestätigt wurden. Wichtig ist, dass sie die Ansichten und die aktuellen Bedürfnisse praktisch tätiger Pathologen aus verschiedenen Lehr- und Kantonsspitälern sowie aus privaten Praxen einbeziehen.

In early 2021, the Swiss Digital Pathology Consortium (SDiPath) initiated a Delphi process to generate best-practice recommendations for various phases of the process of digitization in pathology for the local Swiss environment, encompassing the following four topics: (i) scanners, quality assurance, and validation of scans; (ii) integration of scanners and systems into the pathology laboratory information system; (iii) the digital workflow; and (iv) digital image analysis (DIA)/artificial intelligence (AI). As digital pathology (DP) is increasingly integrated into routine clinical pathological diagnostics by academic and private pathology institutes, setting up national recommendations to standardize procedures and support implementation was perceived as a current need [1–4]. This includes the implementation of DIA algorithms and AI tools, as they have become increasingly attainable and desirable in daily sign out.

Herein, we briefly outline the recommendations related to the validation and use of DIA and AI as it applies to the algorithm-aided extraction of information from histological images to support clinical decision making. The recommendations generated were drafted by a dedicated DIA/AI working group and were subsequently verified by members of SDiPath (*N* = 25) using a Delphi process. All members of SDiPath (*N* > 170) were encouraged to participate. Importantly, the recommendations include the view and the currently perceived needs of practicing pathologists from multiple academic and cantonal hospitals as well as private practices, who comprised 76% of the responding participants. The rest of the participants were scientists, IT personnel, and laboratory staff. We refer the reader to the original guideline publication for an in-depth discussion of the recommendations [5].

## Compliance with general quality guidelines

The SDiPath guidelines affirm that DIA/AI intended for diagnostic use needs to be designed in compliance with current laws and regulatory requirements for medical devices, i.e., only officially certified systems (e.g., IVD-CE certified, FDA-approved) or lab-developed systems properly validated with respect to quality control and quality assurance can be applied. Also, and equally important, it is the board-certified pathologist who determines and is legally responsible for the finally rendered diagnosis. If DIA/AI output is used without being approved by a board-certified pathologist, this is considered off-label use. In line with the existing legal requirement to store all glass slides the diagnoses were generated on, we recommend storing the results of the DIA/AI analysis to allow diagnosis retracing. All steps should be documented in a standard operating procedure (SOP) document.

An internal validation of the system on relevant specimens mirroring the real-world clinical use is recommended, even if the system is officially certified (e.g., IVD-CE certified, FDA-approved) to ensure consistency of performance. Documentation of metadata of the scans, the software used, and the validation protocol as well as the performance of the software (reproducibility, intra-observer variability, and known problems regarding performance) should be provided. When validating the system, a concordance testing needs to be carried out and revalidation shall be performed whenever changes are made in the workflow.

## Workflow considerations

Quality control steps verifying that the scans are suitable for analysis and that all relevant areas have been analyzed are required. User training, personnel, and technical requirements, as well as SOPs for hardware and software malfunctions and management updates, need to be provided. In-depth information about the type and application of the DIA/AI system used in the diagnostic process (including quality control measures and validation steps) needs to be included into the pathology report. There are also multiple technical properties that were agreed on to be paramount. In short, the DIA/AI systems need to be well integrated into the pathology workflow environment and automated, in order to enable streamlining of the diagnostic process in the era of increasing workload.

Importantly, for the pathologist, it is most desirable to have visual control of the algorithm’s results, and this should highlight the regions which determined its output. Understanding how the system is generating output and decisions becomes increasingly more critical as the level of autonomy of these systems will rise in the future. It is suggested that the systems should enable a feedback loop to improve performance.

As a last comment, finding parallels with the multiple comments regarding where “Back to the Future II” was wrong in predicting current everyday life and technology [6, 7], we appreciate that the technological possibilities and standards as well as knowledge in the field of digital pathology are developing rapidly. The current recommendations, published elsewhere in full length [5], have been generated to facilitate current development and implementation and shall be reviewed and updated as technology advances.

## Declarations

For this article no studies with human participants or animals were performed by any of the authors. All studies mentioned were in accordance with the ethical standards indicated in each case.

The supplement containing this article is not sponsored by industry.
